# A Study on the Bond–Slip Relationship of the CFRP–Steel Interface of CFRP Strengthened Steel

**DOI:** 10.3390/ma15124187

**Published:** 2022-06-13

**Authors:** Xinyan Guo, Zuodong Wu, Yi Yang, Jiahao Bai, Qianziyang Zhou

**Affiliations:** School of Civil Engineering and Transportation, South China University of Technology, Guangzhou 510640, China; xyguo@scut.edu.cn (X.G.); 202020107408@mail.scut.edu.cn (Z.W.); u981111@163.com (J.B.); 13535543659@163.com (Q.Z.)

**Keywords:** CFRP, steel plate, double-strap experiment, bond–slip model, bond properties, FE analysis

## Abstract

The bonding interface between the CFRP and the steel plate is the weak link of CFRP-strengthened steel structures. This paper studies the bond–slip relationship of the CFRP–steel interface by experiments and numerical tests. First, a series of double-strap experiments on a CFRP-strengthened steel plate are carried out. The results show that the maximum shear stress of the bonding interface of the Q345B specimen is larger than that of the X100 specimen. The initial slip and maximum slip become larger as the thickness of the bonding interface becomes larger. Finite element analysis of the above tests is carried out; we introduce the maximum stress criterion to simulate the bonding interface, which assumes that when the nominal stress of the material reaches the maximum nominal stress of damage, the material begins to damage. The FE model established has proved very effective for analyzing the bond characteristics of CFRP-strengthened steel plates. Finally, a verification test was carried out, using an FE analysis to verify the accuracy of the modified equations; the results prove that the results of the modified equations are in good agreement with the numerical results and experiment results, which verifies the effectiveness of the equations.

## 1. Introduction

CFRP (carbon fiber reinforcing polymer) has attracted great attention in engineering [1,2] because of its high strength, good corrosion resistance, strong adaptability, and convenient construction [3,4]. The traditional welding methods to repair defects of steel structures affected by the environment and load during service often have problems, such as long construction, difficult maintenance, and residual stress, affecting the structural stability and fatigue [5], but the use of CFRP reinforcement can avoid these problems effectively [6,7]. At present, there are many cases of strengthening steel structures with CFRP. Miller et al. [8] used CFRP to strengthen and repair a steel girder bridge, and Galvez [9] and Marques [10] used CFRP-reinforced joints to strengthen the steel structures on bus and aerospace heat insulation panels, respectively.

Due to the use of adhesive for reinforcement, the bonding interface between CFRP and steel is the weak link of reinforced components [11,12]. The reinforcement effect of CFRP usually depends on the bond performance of the interface. Therefore, many researchers have carried out in-depth studies on the bond performance of the interface of CFRP-strengthened structures. Yu et al. [13] carried out a series of single-strap tensile tests of CFRP-reinforced steel and obtained that the bond–slip curve of linear bind fit the triangular bilinear model, which is a bond–slip model with a triangular shape containing an ascending branch and a descending branch. Sabrina Fawzia et al. [14] pointed out that the thickness of the bonding interface has a significant affection on the bond–slip model, and the initial slip and maximum slip increased with the increase in the thickness of the bonding interface. The initial slip is the slip at the end of the ascending branch, and the maximum slip is the slip at the end of the descending branch. The experiment of Wang et al. [15] showed that the interfacial bond strength gradually increased with the increase in the thickness of the bonding interface, and the interfacial bond strength of different adhesives would be different.

Majidi et al. [16] proposed the point stress method by using finite element analysis to predict the ultimate load of double shear specimens, which can efficiently calculate the ultimate load of double-strap specimens. Li et al. [17] carried out experiments and numerical analysis to study the effects of different types of epoxy adhesive and CFRP; the result showed that different epoxy adhesives or CFRPs with distinct mechanical properties can lead to different failure modes for CFRP–-steel bonding interfaces. Ke et al. [18] proposed a novel film adhesive and conducted experiments on double-lap joints (DLJs) with different bond lengths to prove the validity of the adhesive. The result showed that the bonding interfaces in CFRP–steel composites achieved superior strength, ductility, and high-temperature resistance. The authors’ team has previously conducted a series of double-strap experiments of CFRP-reinforced steel [19]. By measuring the strain of the bonding interface with the strain gauge and DIC (i.e., digital image correlation), the failure characteristics and mechanical behavior of the CFRP-reinforced steel plate were analyzed. It was concluded that the thickness of the bonding interface and steel type affected the load displacement curve, stress distribution, and effective bond length (the critical bond length when the ultimate load of the bonding interface no longer increase with the increase of the bond length) of the specimens.

From the existing literature, it can be seen that there is little research on the influence of steel type on bonding interface. Furthermore, though there exists several bond–slip models to characterize the property of bonding interface, it is difficult to predict it. In this paper, a series of supplementary experiments were carried out to study the bond–slip model of the CFRP–steel interface based on the previous tests [19]. Three existing bond–slip model theories were compared, and a set of modified equations according to the test results was given. A finite element numerical analysis was also carried out to verify the accuracy of the expressions. The modified equations have a good prediction effect on the property of bonding interface.

## 2. Double-Strap Experiment

**Figure 1:**
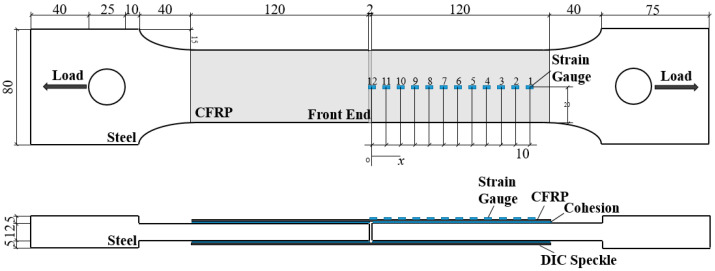
Specimen Size and Test Point Distribution.

Figure 1 shows the double-strap joint used in this test; the fabrication of specimens follows the standards ASTM E8/E8M-16a [20] and ASTM 3528-96(2016) [21]. The double-strap specimens are designed to accurately measure the shear stress of bonding interface by reducing moment of bonding interface as much as possible. The steel plate was processed by wire cutting, and the surface reinforced by CFRP was polished and cleaned with acetone. The CFRP was woven by carbon fiber and prepreg mixed with epoxy resin and a curing agent. The carbon fiber used was T700-12k, manufactured by Toray Company (Tokyo, Japan). The epoxy resin adhesive (lica-131), mixed with small steel balls of uniform size to control the thickness of the bonding interface, was applied on the surface of the steel plate. The strain of the specimen was measured by strain gauge and DIC. The two types of steel used in the test were Q345B and X100. In the test, the CFRP material used to strengthen the steel plate was made by t700-12k, produced by the Toray company from Japan; the adhesive used was lica-131. Table 1 shows the mechanical properties of materials used in this test.

**Figure 2:**
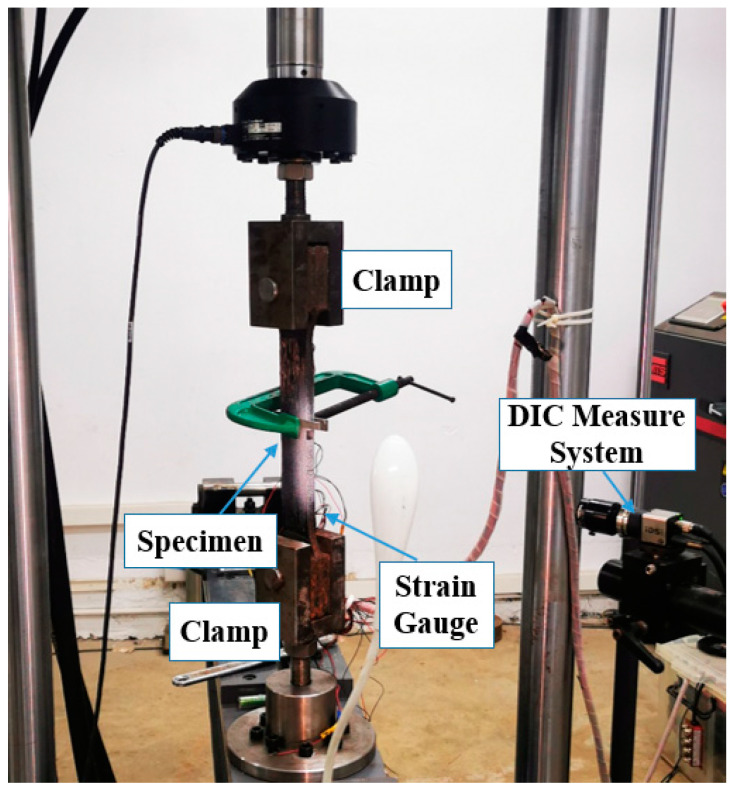
Test Equipment.

Specimens in this test were divided into six groups, with three specimens in each group. Specimens with two different steel types (Q345B and X100) adopted three different thicknesses (0.1 mm, 0.5 mm, and 0.8 mm) of bonding interface, respectively. Table 2 shows a specific configuration of the specimen groups. An MTS servo testing machine was used for the test device, and the loading rate was set at 0.005 mm/s. The loading device is shown in Figure 2.

A previous study [22] indicated that galvanic corrosion could occur when steel has direct contact with CFRP material in aggressive environments. However, this phenomenon can be avoided by increasing the thickness of the adhesive layer and improving the plumpness of the adhesive layer. In the past experiments on CFRP–steel composite, galvanic corrosion has never occurred.

## 3. Test Results

The bond–slip curve, i.e., the shear stress–slip curve of the bonding interface, could characterize the load-carrying process and failure process of the interface as well as the macroscopic bearing performance of the interface. The previous test [19] pointed out that the maximum shear stress of the bonding interface was affected by the thickness of the bonding interface and the steel type. According to the experiment in Section 1, this paper analyzed the bond–slip curve of the bonding interface and the effect caused by the thickness of the bonding interface and the steel type. The shear stress and slip at the midpoint of test point *i* and test point *i* − 1 are given by:

(1) $$\begin{array}{l} \tau_{i}=\frac{\Delta \varepsilon_{i}E_{p}t_{p}}{\Delta l_{i}}=\frac{\left(\varepsilon_{i}-\varepsilon_{i-1}\right)E_{p}t_{p}}{l_{i}-l_{i-1}}\\ s_{i}=\sum_{j=1}^{i}\frac{\varepsilon_{j}+\varepsilon_{j-1}}{2}\Delta l_{j} \end{array}$$

where $\tau_{i}$ is the shear stress at test point *i*, $l_{i}$ is the distance between test point *i* and the front end of the steel plate, $\Delta l_{i}$ is the distance between test point *i* and test point *i* − 1; $E_{p}$ and $t_{p}$ are the elastic modulus and thickness of CFRP, respectively. This paper took as the test point 25 mm from the front end of the steel plate to plot the bond–slip curve, for this point experienced the complete process of the shear stress fluctuation and transmission.

**Figure 3:**
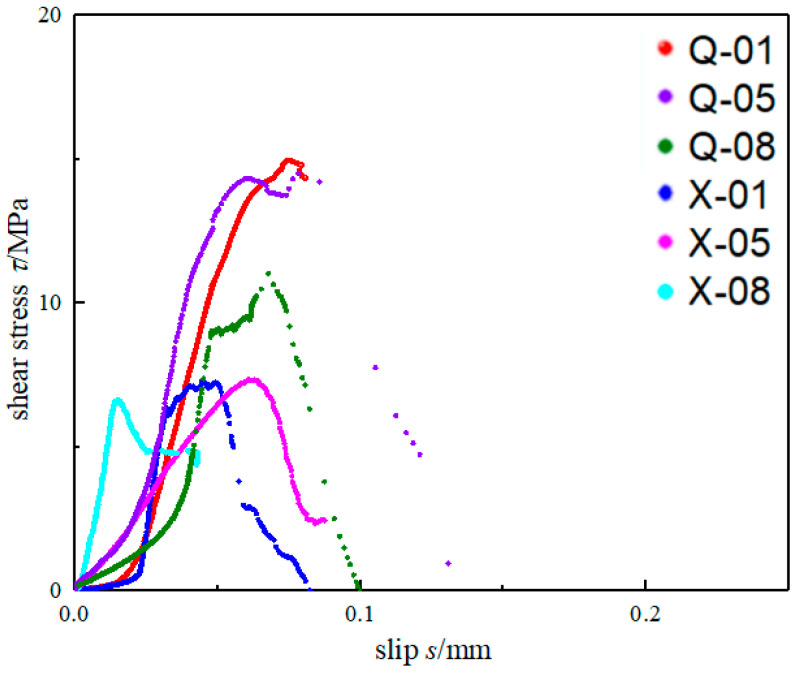
Bond–slip curves.

Six bond–slip curves of the bonding interface of each test group are plotted in Figure 3 according to the shear stress and slip calculated by Equation (1). It can be seen that for all six groups of specimens, the bond–slip relationship approximated to the bilinear bond–slip model as containing an ascending branch and a descending branch. At the same time, for the same steel type, the $\tau_{\max}$ of the interface was similar: for Q345B specimens, the $\tau_{\max}$ of the interface for Q-01 and Q-05 was similar, and the $\tau_{\max}$ for Q-08 is about 24% less than those of the two previous specimens; the $\tau_{\max}$ was similar for all X100 specimens. As for different steel type, the $\tau_{\max}$ of the Q345B specimens was larger than that of the X100, which was about 1.9 times of X100. It can be seen that the type of steel effect on the bond–slip model dramatically, which represent in the maximum shear stress ($\tau_{\max}$) and interfacial facture energy ($G_{f}$) of Q345B being much bigger than that of X100, it may be due to the difference in surface roughness of different steel types. A steel plate with higher strength may decrease the bond property of the bonding interface, which awaits further study. With the increase in the thickness of the bonding interface, the initial slip ($\delta_{1}$) changes little, yet the maximum slip ($\delta_{f}$) of the interface decreases.

## 4. Comparison of Bond–Slip Models

**Figure 4:**
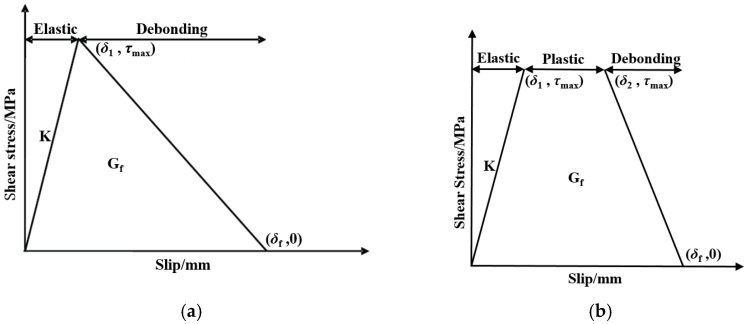
Sketch of two kinds of bond–slip model. (**a**) Bilinear bond–slip model; (**b**) Trapezoidal bond–slip model.

For different adhesives with different mechanical properties, there exist two different bond–slip models [12,22]: the bond–slip model of a linear adhesive can be approximated by a bilinear curve (Figure 4a), while a nonlinear adhesive can be approximated by a trapezoidal curve (Figure 4b). For the bilinear bond–slip model, in the elastic stage, the shear stress increased linearly with the increase in slip, and the growth rate between the shear stress and slip is the interface stiffness K; when the shear stress reached the maximum shear stress $\tau_{\max}$ (the slip reaches $\delta_{1}$), the interface entered the debonding stage, and the shear stress decreased linearly with the slip. At the end of the descending branch, the shear stress decreased to 0 as the slip reaches $\delta_{f}$. The trapezoidal bond–slip model has an additional platform before the debonding stage, in which the shear stress remains unchanged.

At present, the linear adhesive is mostly studied. Many researchers have carried out experimental research and provided fitting equations for bond–slip parameters. Xia and Teng [23] proposed that the bond–slip curve of the CFRP-strengthened steel specimens damaged by debonding of the bonding interface was very close to a bilinear shape and established a simplified bilinear bond–slip model. Fernando [24] provided a bilinear bond–slip model fitting equation through the experiment and finite element simulation of several different adhesives; Fernando believed that the result was more accurate than Xia’s. Fawzia [14] believes that $\delta_{f}$ will increase significantly when the thickness of the bonding interface increases to more than 0.5 mm. Table 3 gives the expressions of the above three bilinear bond–slip models.

**Figure 5:**
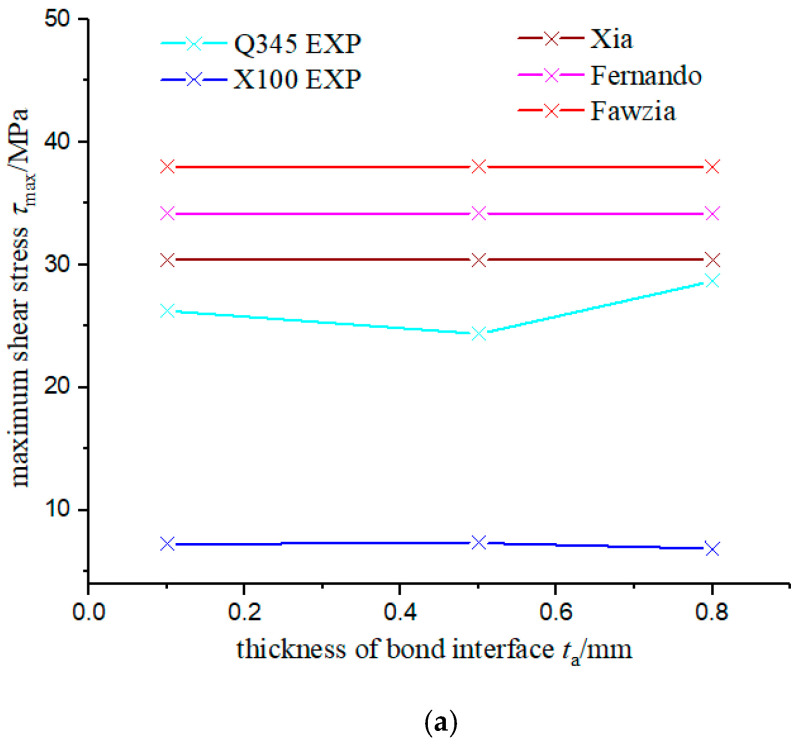

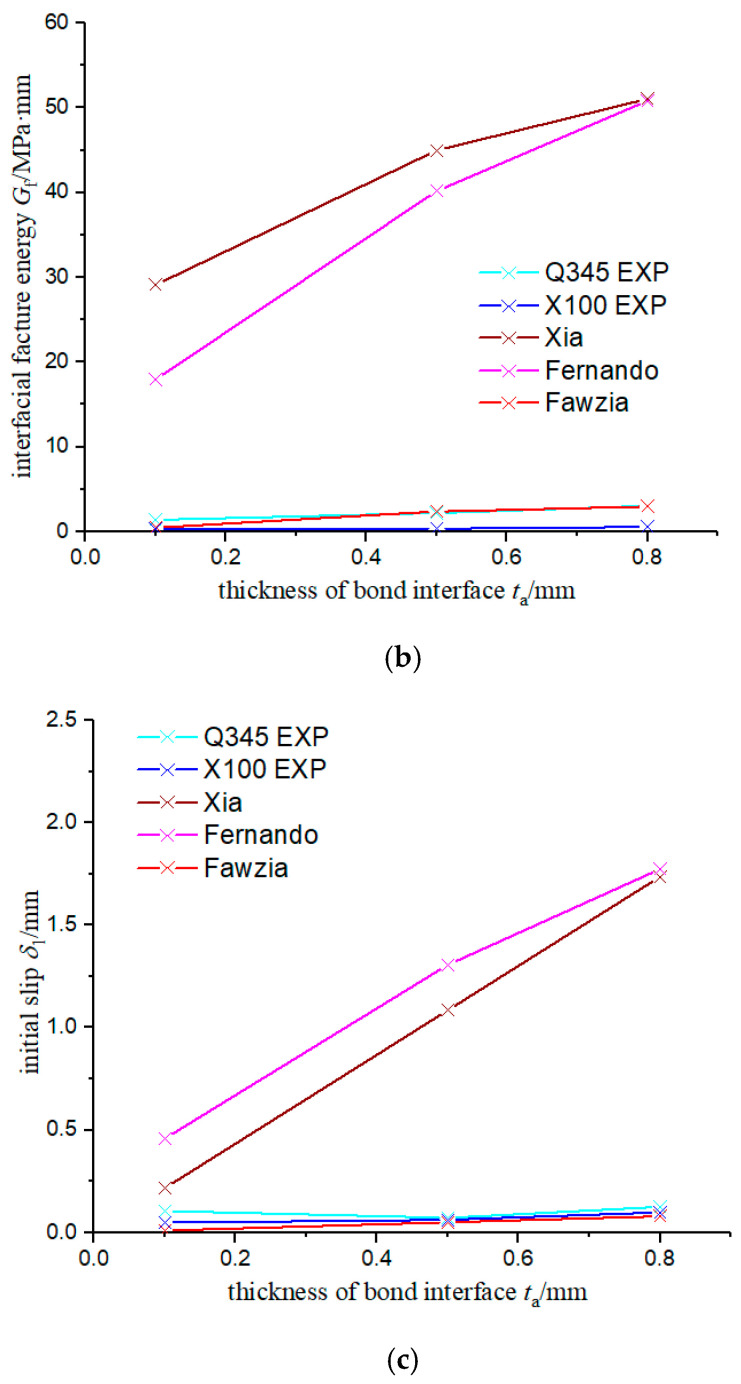
Comparison of Calculation Results of Three Bond–Slip Models and the Experimental Result. (**a**) Maximum shear stress. (**b**) Interfacial facture energy. (**c**) Initial slip.

Figure 5 shows the comparison of calculation result of the three bond–slip models and experimental results. Combined with Figure 3, it can be seen that among the $\tau_{\max}$ given by the three models, the change in the thickness of the bonding interface did not affect the value of $\tau_{\max}$, which was consistent with the experimental results, where the Xia model was the closest to the experimental results of Q345; for $G_{f}$ and $\delta_{1}$, the fitting results given by Fawzia were close to the experimental results, while the fitting results of Xia and Fernando were 14~20 times larger than the experimental results, but the trend of bond–slip parameters fit well between Xia’s model and the experimental result. Moreover, for the X100 specimen, only the $\delta_{1}$ given by Fawzia was in good agreement with the experimental results.

According to the experimental results in Section 2, although the fitting results of the Xia model were quite different from the experimental results, the trend of the parameters with the thickness of bonding interface was very similar to the experimental results. Therefore, the coefficients of the Xia model were used to better fit the bond–slip model of the bonding interface of the CFRP-reinforced steel using Q345 and X100; the modified fitting equations are given as follow:

Q345B:

(2) $$\begin{array}{l} \tau_{\max}=0.7\sigma_{\max}\\ G_{f}=0.16{\left(\frac{\sigma_{\max}}{G_{a}}\right)}^{0.56}t_{a}^{0.3}\\ \delta_{1}=\frac{\tau_{f}t_{a}}{14G_{a}} \end{array}$$

X100:

(3) $$\begin{array}{l} \tau_{\max}=0.19\sigma_{\max}\\ G_{f}=0.3{\left(\frac{\sigma_{\max}}{G_{a}}\right)}^{0.56}t_{a}^{0.3}\\ \delta_{1}=\frac{\tau_{f}t_{a}}{16G_{a}} \end{array}$$

## 5. Numerical Simulation Analysis

### 5.1. Finite Element Model

**Figure 6:**
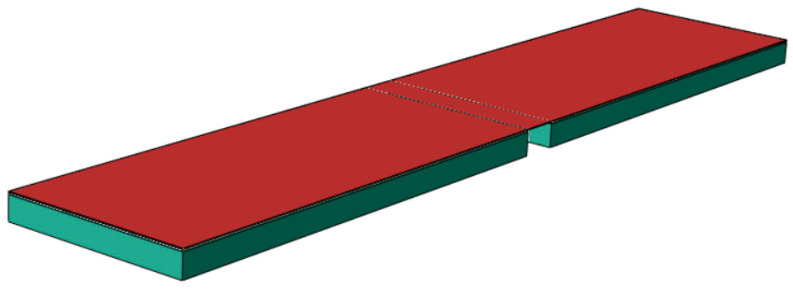
Finite element model.

In this paper, the finite element model of the CFRP-reinforced steel plate was established by the finite element analysis software ABAQUS, and the numerical analysis was made based on the FE model. In the FE model, the C3D8R element was used for the steel plate and the CFRP. To accurately simulate the bond characteristics of the bonding interface, a three-dimensional cohesive element COH3D was used for the bonding interface. In order to reduce the calculation scale, taking the neutral plane in the thickness direction of the steel plate as the symmetrical plane, a 1/2 model of the double-strap test specimen was established, constraints in the X, Y, and Z directions were applied to the nodes on the free end and the symmetrical plane, and the horizontal displacement was applied to the loading end of the steel plate to simulate the loading process. The model consisted of 3605 C3D8R units, 3000 COH3D units, and 13,996 nodes; Figure 6 shows the FE model established in ABAQUS.

The modeling of the cohesive element of the bonding interface needs to introduce the traction–separation criterion to simulate the bond characteristics. The stiffness and nominal stress in three directions (one normal direction and two tangential directions) need to be input as material parameters. Its relationship can be represented by:

(4) $$\left\{\begin{array}{l} t_{n}\\ t_{s}\\ t_{t} \end{array}\right\}=\left[\begin{array}{ccc} K_{\mathrm{nn}} & 0 & 0\\ 0 & K_{\mathrm{ss}} & 0\\ 0 & 0 & K_{\mathrm{tt}} \end{array}\right]\left\{\begin{array}{l} \varepsilon_{n}\\ \varepsilon_{s}\\ \varepsilon_{t} \end{array}\right\}$$

where $t_{n}$, $t_{s}$, and $t_{t}$ are the nominal stress in the normal direction and tangential directions, respectively; $K_{\mathrm{nn}}$, $K_{\mathrm{ss}}$, $K_{\mathrm{tt}}$ and $\varepsilon_{n}$, $\varepsilon_{s}$, $\varepsilon_{t}$ are the respective stiffness and the nominal strain in three directions. The bond–slip parameters (stiffness *K*, maximum shear stress $\tau_{f}$, and interface facture energy $G_{f}$) from Section 3 were substituted into the bilinear traction–separation criterion.

The initial damage criterion corresponds to the critical damage condition of cohesive materials. The traction–separation criterion included the maximum stress criterion, maximum strain criterion, secondary stress criterion, and secondary strain criterion. In this paper, the maximum stress criterion was adopted; that is, when the nominal stress of the material reached the maximum nominal stress of damage, the material began to damage, which can be represented by:

(5) $$\mathrm{Max}\left\{\frac{t_{n}}{t_{n}^{0}},\frac{t_{s}}{t_{s}^{0}},\frac{t_{t}}{t_{t}^{0}}\right\}=1$$

where $t_{n}^{0}$, $t_{s}^{0}$, and $t_{t}^{0}$ are the respective maximum nominal stress of damage in three directions.

### 5.2. Numerical Simulation Results

Table 4 shows the comparison of the ultimate load *P*_ult_ between the experiment results and the finite element simulation results. It can be seen that the error of *P*_ult_ between the test results and the finite element results was within 10%.

**Figure 7:**
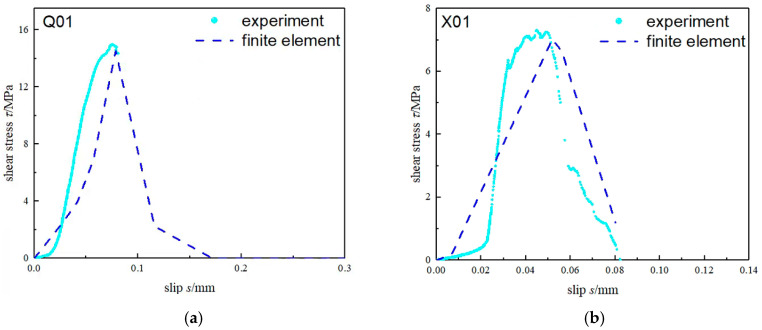

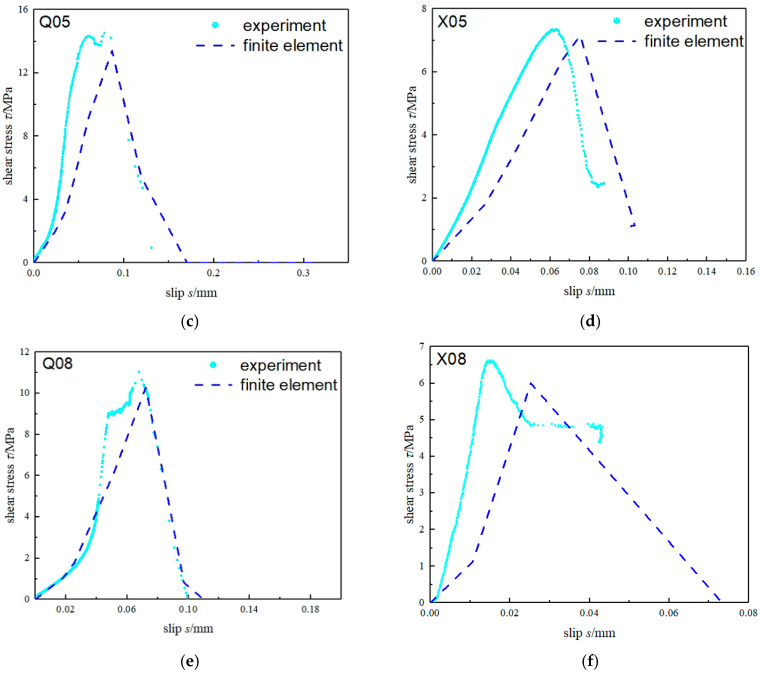
Comparison of Bond–Slip Curve between Different Groups. (**a**) Q-01; (**b**) X-01; (**c**) Q-05; (**d**) X-05; (**e**) Q-08; (**f**) X-08.

Figure 7 shows the comparison between the test results and the numerical simulation results of the bond–slip curves of specimens with different steel and bonding interface thickness. The results show that the total error between the numerical simulation results of the bond–slip curves and the test results was also less than 10%. Therefore, the finite element model could accurately simulate the bond characteristics of the CFRP-reinforced steel specimens. This FE model can be effectively used to analyze the interfacial bond properties of CFRP-strengthened steel specimens.

## 6. Verification Test

In order to further verify the validity of Equations (2) and (3) given in Section 3, a verification test was carried out in this paper. In this verification test, six specimens of CFRP-strengthened steel plate using Q345B and X100 (each for three) with a bonding interface thickness of 1.0 mm were used. All test conditions were exactly the same as those in Section 1 except the thickness of the bonding interface. At the same time, the finite element model established in Section 4 was used to simulate the verification test.

**Figure 8:**
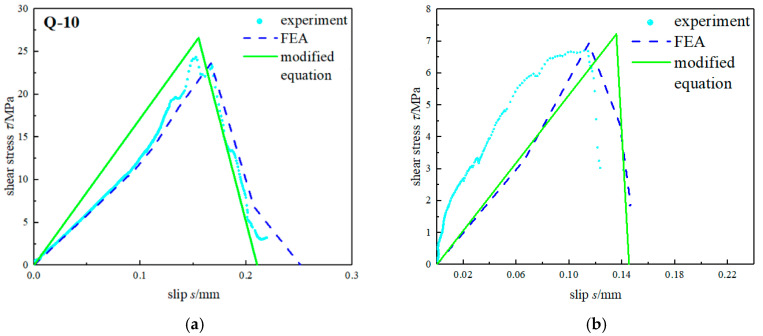
Comparison of Bond–Slip Curve between the Modified Equations, Verification Test, and FEA. (**a**) Q-01; (**b**) X-10.

The bond–slip curve obtained by the verification test, modified equation, and FE analysis are given in Figure 8. It can be seen that the bond–slip parameters obtained by the modified equations were in good agreement with the verification test results and finite element results, with no error. Therefore, the fitting formulas for the bond–slip model given by Equations (2) and (3) well predicted the bond characteristics of the CFRP–steel interface.

## 7. Conclusions

This paper studied the bond properties of CFRP-strengthened steel plates by experiments and simulation. Three existing bond–slip models were analyzed, and a set of modified fitting equations for bond–slip parameters were proposed and verified. The main conclusions are as follows:

1. The $\tau_{\max}$ of the bonding interface of the CFRP-reinforced steel plate was not affected by the change in the thickness of the bonding interface, but the $\tau_{\max}$ of the Q345B specimen was larger than that of the X100, about 1.9 times that of the X100. It may be because of the difference of surface roughness between two steel types. A steel plate with higher strength may decrease the bond property of the bond interface, which awaits further study.
2. The result of $\tau_{\max}$ given by Xia’s model was the closest to the experimental result of Q345; for the $G_{f}$ and $\delta_{1}$, Fawzia’s fitting results were the closest to the experimental results, but Xia’s model better predicted the trend in the thickness of the bonding interface on the bond–slip parameters. The initial facture energy of bonding interface is linear correlate with thickness of bonding interface, and initial slip is exponential correlated with thickness of bonding interface.
3. An FE model for CFRP-strengthened steel was established; the model proved to be effective for the analysis of the bond characteristics of the CFRP–steel interface, for the total error between the numerical simulation results of the bond–slip curves and the test results was less than 10%. Furthermore, the introduction of the maximum stress criterion proved to be very helpful to the modeling of the bonding interface.
4. A set of modified equations for the bond–slip parameters was given. The effectiveness of the modified equations for predicting the bond characteristics of the CFRP–steel interface was verified by means of a verification test and FE analysis. The modified equations have a good prediction effect on the property of bonding interface, and the FE model and modified equations established in this study can be extended to further research, such as fatigue performance, crack propagation, and mechanical property under the specific environment of CFRP-strengthened steel structures.

## Figures and Tables

**Table 1:** Mechanical Properties of Materials in this Experiment.

	Elastic Modulus (GPa)	Poisson Ratio	Yield Strength (MPa)	Tensile Strength (MPa)	Shear Strength (MPa)
Q345B	206	0.3	345	455	/
X100	210	0.3	700	790	/
CFRP	230	0.307	/	4750	/
Lica-131	2.4	/	/	38	14

**Table 2:** Test Configuration and Specimen Groups.

Steel Type	Test Groups	Thickness of Bonding Interface (mm)	Sample
Q345B	Q-01	0.1	3
	Q-05	0.5	3
	Q-08	0.8	3
X100	X-01	0.1	3
	X-05	0.5	3
	X-08	0.8	3

**Table 3:** Expressions of Parameters of Different Bond–Slip Models.

Xia [23]	Fernando [24]	Fawzia [14]
$\begin{array}{l} \tau_{\max}=0.8\sigma_{\max}\\ G_{f}=31{\left(\frac{\sigma_{\max}}{G_{a}}\right)}^{0.56}t_{a}^{0.27}\\ \delta_{1}=\frac{\tau_{f}t_{a}}{G_{a}} \end{array}$	$\begin{array}{l} \tau_{\max}=0.9\sigma_{\max}\\ G_{f}=628t_{a}^{0.5}R^{2}\\ \delta_{1}=0.3{\left(\frac{t_{a}}{G_{a}}\right)}^{0.65}\sigma_{\max} \end{array}$	$\begin{array}{l} \tau_{\max}=\sigma_{a}\\ \delta_{1}=\frac{t_{a}}{10}\\ \delta_{f}=\frac{t_{a}}{4}\;\;\mathrm{for}\;\;t_{a}=0.1-0.5\mathrm{mm}\\ \delta_{f}=0.125+\frac{t_{a}-0.5}{10}\;\;\mathrm{for}\;\;t_{a}=0.5-1\mathrm{mm} \end{array}$

where $\sigma_{\max}$ and $G_{a}$ are the tensile strength and the shear strength of the adhesive, *t*_a_ is the thickness of bonding interface, and *R* is the tensile strain energy of the adhesive.

**Table 4:** Comparison of *P*_ult_ between Experiment results and FE results.

Test Groups	EXP	FEA
Q-01	56.8	51.6
Q-05	56.3	52.9
Q-08	41.7	39.0
X-01	25.3	28.9
X-05	25.6	31.7
X-08	18.8	25.4

## Data Availability

The data presented in this study are available on request from the corresponding author.
